# PRR11 and SKA2 promote the proliferation, migration and invasion of esophageal carcinoma cells

**DOI:** 10.3892/ol.2020.11615

**Published:** 2020-05-13

**Authors:** Jie Chen, Hong-Mei Yang, Hui-Chong Zhou, Rui-Rui Peng, Zhao-Xiao Niu, Chun-Yan Kang

**Affiliations:** 1 Department of Pathophysiology, Henan Medical College, Zhengzhou, Henan 450000, P.R. China; 2 Department of Gastroenterology, Second Affiliated Hospital of Zhengzhou University, Zhengzhou, Henan 450000, P.R. China

**Keywords:** PRR11, SKA2, esophageal carcinoma, progression

## Abstract

Proline-rich protein 11 (PRR11) together with its upstream adjacent gene, spindle and kinetochore associated 2 (SKA2), represent a classic, head-to-head gene pair. The role of the PRR11 and SKA2 gene pair has been described in various types of cancer, including breast cancer, non-small cell lung cancer, hepatocellular carcinoma and ovarian carcinoma. However, its role in esophageal carcinoma (ESCC) remains unclear. The mRNA expression levels of PRR11 and SKA2 were examined in ESCC surgical specimens. In addition, the role of PRR11 and SKA2 in the proliferation and migratory and invasive capacities of EC9706 and EC109 cell lines was examined. The results from the present study demonstrated that PRR11 and SKA2 expression levels were upregulated in ESCC tissues compared with adjacent normal tissues. Furthermore, PRRl1 and SKA2 knockdown significantly inhibited the proliferation and migratory and invasive capacities of ESCC cells. Conversely, PRRl1 and SKA2 overexpression significantly promoted the proliferation and migratory and invasive capacities of ESCC cell lines via activation of the AKT signaling pathway and certain markers of epithelial-mesenchymal transition, including Snail and N-cadherin. The results from the present study suggested that the PRR11 and SKA2 gene pair may represent a potential target in the diagnosis and treatment of ESCC.

## Introduction

Esophageal squamous cell carcinoma (ESCC), which is one of the most common types of cancer, was the sixth leading cause of cancer-associated mortality in China in 2016 (1). In 2012, an estimated 455,800 new esophageal cancer cases and 400,200 deaths were reported (2). Although surgery and radiation therapy are effective therapeutic strategies for certain tumors diagnosed at an early stage, a number of patients eventually progress to advanced stages of cancer (3). Based on previous epidemiologic studies, it is widely accepted that genetics serve a crucial role in the development and progression of ESCC (4,5); however, the underlying mechanisms remain unclear. It is therefore important to fully understand the development and progression of ESCC, in order to identify novel therapeutic targets.

The proline-rich protein 11 (PRR11) gene is located on chromosome 17q22 and contains a bivalent nuclear localization signal, two proline-rich regions and a zinc finger domains (6). In recent years, PRR11 has been reported to serve as a candidate oncogene in various types of cancer, including pancreatic cancer, breast cancer, non-small cell lung cancer, hepatocellular carcinoma and ovarian carcinoma (7–9). PRR11 and its upstream adjacent gene, spindle and kinetochore associated 2 (SKA2), are a classic head-to-head gene pair, driven by a prototypical and bidirectional promoter (10). SKA2, which is involved in the formation of the Ska complex, is involved in the maintenance of the mitotic mid-plateau and shut-down of the spindle checkpoint (11–14). Currently, studies on PRR11 and SKA2 mainly focus on lung and breast cancer (7,8,10); however, the role of PRR11 and SKA2 in ESCC remains unclear.

The present study evaluated the importance of PRR11 and SKA2 in the progression of ESCC, and demonstrated that the expression level of these genes was highly expressed in human ESCC tissues. Furthermore, the present study revealed that PRR11 and SKA2 serve important roles in the proliferation and migratory and invasive capacities of ESCC cells *in vitro*. In addition, the underlying mechanism of PRR11 and SKA2 in the progression of ESCC was investigated.

## Materials and methods

### 

#### ESCC tissue samples

A total of 30 pairs of ESCC and adjacent normal tissues were obtained from patients following resection surgery at the Second Affiliated Hospital of Zhengzhou University between January 2014 and December 2017. The adjacent normal tissue <1-cm away from the tumor tissue. The median age was 52 (41–61) years old, and there were 22 males and 8 females. Some clinical parameters, such as sex, age, history of drinking and smoking, tumor site, TNM staging (the 8th edition) (15), and tumor differentiation. All patients provided informed signed consent. The specimens were immediately stored at −80°C until RNA extraction. The present study was approved by the Ethics Committee of the Second Affiliated Hospital of Zhengzhou University.

#### RNA preparation and reverse transcription-quantitative polymerase chain reaction analysis

Total RNA was extracted from the 30 paired ESCC tumor tissues and adjacent normal tissues using TRIzol^®^ reagent (Invitrogen; Thermo Fisher Scientific, Inc.) according to the manufacturer's instructions, and 2 µg RNA was reverse transcribed to cDNA using the Reverse Transcription System (A3500, Promega Corporation) according to the manufacturers' instructions. The mRNA level was detected using a PikoReal 96 Real-Time PCR system (Thermo Fisher Scientific, Inc.) and SYBR^®^ Premix Ex Taq (Takara Bio, Inc.). The thermal cycling conditions were as follows: 40 cycles at 95°C for 20 sec, 60°C for 30 sec, and 72°C for 30 sec, and one cycle of 72°C for 10 min. The sequences of the primers were as follows: PRR11, forward 5′-GAAGCTGGCTAACATCATCCTG-3′, reverse 5′-CTCTGGGTTATGCAGTTCTGG-3′; SKA2, forward 5′-GGAACTGATGTTCCAGAAAGCTG-3′, reverse 5′-AGCTCCAGGTCTGTTTGCTT-3′; and GAPDH, forward 5′-ATGACCCCTTCATTGACCTCA-3′ and reverse 5′-GAGATGATGACCCTTTTGGCT-3′. The relative mRNA expression level in each sample was calculated using the comparative expression level 2^−∆∆Cq^ method (16).

#### Cell culture

The esophageal squamous cell carcinoma cell lines EC9706 and EC109, and 293T cell line were purchased from The Cell Bank of Type Culture Collection of the Chinese Academy of Sciences. Cells were cultured in Dulbecco's Modified Eagle Medium (DMEM) supplemented with 100 U/ml penicillin, 100 mg/ml streptomycin, and 10% (v/v) fetal bovine serum (FBS; Invitrogen; Thermo Fisher Scientific, Inc.) at 37°C in a humidified atmosphere containing 5% CO_2_.

#### Plasmids and cell transfection

A PRR11 and SKA2 coding sequence was constructed and inserted into a pcDNA3.1-Myc plasmid (Invitrogen; Thermo Fisher Scientific, Inc.) to generate the PRR11 and SKA2 overexpression vector. Lentiviral supernatants were produced using the Lenti-X HTX packaging system (Clontech Laboratories, Inc.) according to the manufacturer's protocol and used for transfection of EC109 cells. For negative controls, EC109 cell were transfected with supernatants from empty vectors. A total of 30 µl lentiviral particles (5×10^7^ UT/ml) were suspended in DMEM, and incubated with the EC109 cells (1×10^6^) in a 6-well plate for 12 h until they reached 70% confluence. Next, transfected cells were screened by green fluorescence using flow cytometry (IX-71; Olympus Corporation). Lentiviral short hairpin (sh)RNA targeting PRR11 and SKA2 were designed using software provided by Qiagen, Inc. and inserted into a pLKO.1-TRC vector (Han Heng Biotechnology Co., Ltd.). The lentivirus was packaged in 293T cells and collected following centrifugation at 4°C and 1,000 × g for 2 h. A total of 50 µl lentiviral particles (1×10^8^ UT/ml) were suspended in DMEM, and incubated with the EC9706 cells (1×10^6^) in a 6-well plate for 8 h until they reached 60% confluence. Positive EC9706 cells were selected by puromycin (4 µg/ml) (Han Heng Biotechnology Co., Ltd.) for at least 3 days or sorted by flow cytometry. Subsequent experiments were conducted at 48 h post-transfection. The expression of PRR11 and SKA2 in the resistant clones was further confirmed by western blotting. The sequences of the shRNAs were as follows: shPRR11, forward: 5′-CCGGCCAGAGTTTAGAAGTATTGAACTCGAGTTCAATACTTCTAAACTCTGGTTTTTG-3′ and reverse: 5′-AATTCAAAAACCAGAGTTTAGAAGTATTGAACTCGAGTTCAATACTTCTAAACTCTGG-3′; shSKA2, forward: 5′-CCGGCAAACTTTGTATGCCCGCTTTCTCGAGAAAGCGGGCATACAAAGTTGTTTTTG-3′ and revere 5′-AATTCAAAAACAAACTTTGTATGCCCGCTTTCTCGAGAAAGCGGGCATACAAAGTTTG-3′; and shcontrol, forward: 5′-TGACAAGTGGAACCAGAT-3′ and reverse: 5′-TGTCCCCACTCACGAAGG-3′.

#### Western blotting

EC9706 and EC109 cells were lysed in RIPA lysis buffer (Thermo Fisher Scientific, Inc.) for at least 30 min on ice. The lysates were centrifuged at 10,000 × g for 20 min at 4°C. Protein concentration was determined using Bradford reagent (Sigma-Aldrich; Merck KGaA) according to the manufacturer's instructions. Proteins (10 µg/lane) were separated by 10% SDS-PAGE and transferred onto polyvinylidene fluoride membranes. Then, membranes were blocked with 5% fat-free milk for 1 h at room temperature. Next, membranes were incubated with primary antibodies at 4°C overnight. The primary antibodies were as follows: PRR11 (1:500; cat. no. 3220; Cell Signaling Technology, Inc.), SKA2 (1:500; cat. no. 2419; Cell Signaling Technology, Inc.), phosphorylated (p) and total Akt (1:1,000; cat. no. 10176-2-AP; ProteinTech Group, Inc.), proliferating cell nuclear antigen (PCNA; 1:1,000; cat. no. 10205-2-AP; ProteinTech Group, Inc.), Snail (1:1,000; cat. no. 13099-1-AP; ProteinTech Group, Inc.), Cyclin D1 (1:1,000; cat. no. 60186-1-Ig; ProteinTech Group, Inc.), N-cadherin (1:1,000; cat. no. 22018-1-AP; ProteinTech Group, Inc.) and GAPDH (1:1,000; cat. no. 60004-1-Ig; ProteinTech Group, Inc.). Snail and N-cadherin are markers of EMT, and cyclin D1 is a marker of the cell cycle (17,18). Membranes were then incubated with anti-rabbit horseradish peroxidase-conjugated secondary antibody (cat. no. 7074; 1:2,000; Sangon Biotech Co., Ltd.) for 2 h at room temperature. The immunoreactive protein bands were visualized using an enhanced chemiluminescence kit (Pierce; Thermo Fisher Scientific, Inc.) and a Gel Dox XR system (Bio-Rad Laboratories, Inc.).

#### Crystal violet assay

Control and transfected cells were seeded into 6-well plates at a density of 1,000 cells/well. Cells were cultured in DMEM with 10% FBS and medium was changed every three days. After two weeks of culture, the culture medium was removed and cells were stained with crystal violet (1 ml 0.5% crystal violet solution in 20% methanol) for 10 min at room temperature. Cells were washed with PBS and images were captured using a digital camera. The optical density (OD) value was measured at 600 nm with a microplate reader.

#### Cell proliferation assay

EC9706 and EC109 cells were seeded in 96-well plates at a density of 1,000 per well in triplicate. MTT solution (20 µl; 5 mg/ml) (APExBIO Technology LLC) was added to the medium and incubated at 37°C for 4 h. The medium was removed, and 200 µl DMSO was added to dissolve the formazan crystals. The absorbance was measured using an automatic microplate reader at a wavelength of 490 nm.

#### Boyden chamber assay

A total of 2×10^5^ EC9706 and EC109 cells were loaded into the upper chamber of a 10-well Boyden Chamber (Neuro Probe, Gaithersburg, MD, USA) with a polycarbonate membrane of 8 µm pore sizein 200 µl of medium containing 1% FBS. Medium (250 µl) containing 10% FBS was added to the bottom well of the chamber. Cells were incubated for at 37°C for 6 h. Cells that have migrated through the pores into the lower chamber were stained with hematoxylin and eosin for 10 min at room temperature. Cells were then then photographed using an inverted microscope (OLYMPUS) at magnification, ×400. Cells were counted from four randomly selected fields by ImageJ software (version 1.8.0, National Institutes of Health). Experiments were repeated in triplicate.

#### Transwell assay

ESCC cell invasion was examined using a Transwell assay with polyethylene terephthalate membranes (24-well inserts; 8.0 µm; Corning Inc.). Cell suspension (150 µl) containing 2×10^5^ cells was loaded into the upper well that was precoated with 20% Matrigel (BD Biosciences) at 37°C for 3 h. DMEM (500 µl) containing 10% FBS was added to the bottom of the well. After 48 h, cells that have invaded the bottom of the membrane were stained with 0.1% crystal violet for 10 min at room temperature and then photographed using an inverted light microscope (Olympus Corporation) at 400× magnification Cells were counted from four randomly selected fields by ImageJ software (version 1.8.0, National Institutes of Health). Experiments were conducted in three independent experiments.

#### Statistical analysis

Data are presented as the mean ± standard deviation and statistical analyses were performed using GraphPad Prism software (version 5; GraphPad Software, Inc.). PRR11 and SKA2 mRNA levels between tumor and adjacent normal tissue groups were analyzed using the paired Student's t-test. PRR11 and SKA2 mRNA levels in the cell lines were determined using an unpaired Student's t-test. Comparisons of multiple groups were analyzed using the ANOVA followed by Tukey's post hoc test. P<0.05 was considered to indicate a statistically significant difference.

## Results

### 

#### PRR11 and SKA2 are upregulated in ESCC tissues

**Figure 1.**
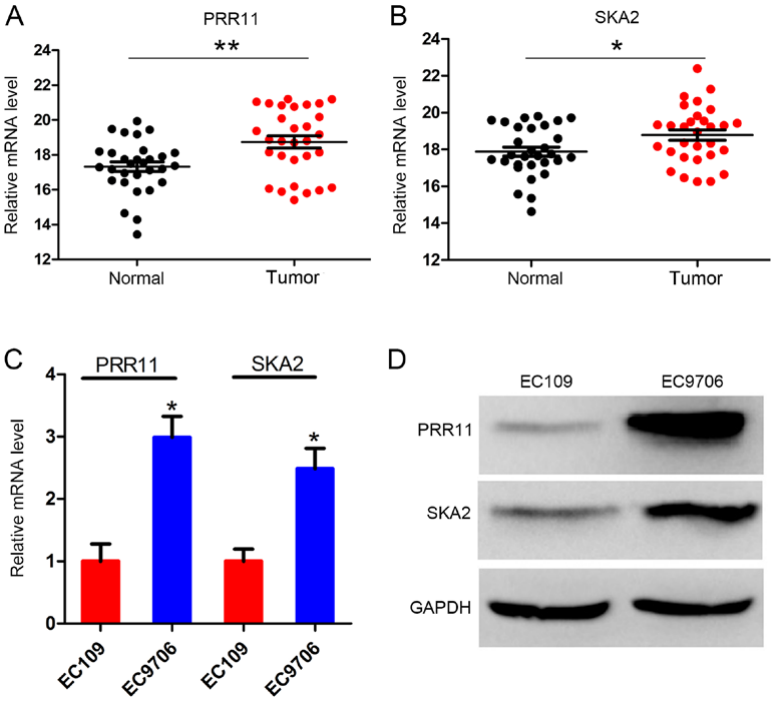
The expression of PRR11 and SKA2 in ESCC tissues and cell lines. (A) PRR11 mRNA levels in 30 pairs of tumor samples and matched normal tissues determined by RT-qPCR. (B) SKA2 mRNA levels in 30 pairs of tumor samples and matched normal tissues determined by RT-qPCR. (C) PRR11 and SKA2 mRNA expression in the two ESCC cell lines EC109 and EC9706. (D) Protein expression of PRR11 and SKA2 in the two ESCC cell lines EC109 and EC9706 by western blotting. GAPDH was used as a loading control. *P<0.05 and **P<0.01. PRR11, proline-rich protein 11; RT-qPCR, real-time quantitative PCR; SKA2, spindle and kinetochore associated 2.

The primary focus of the present study was the expression level of PRR11 and SKA2 in the 30 pairs of ESCC and adjacent normal tissues. The clinicopathological characteristics of the 30 patients are presented in Table I. The results demonstrated that PRR11 expression level was significantly increased in ESCC tissues compared with the adjacent normal tissues (P<0.001; Fig. 1A). Similarly, SKA2 expression level was significantly increased in tumor tissues compared with normal tissues (P<0.05; Fig. 1B). Furthermore, the mRNA and protein levels of PRR11 and SKA2 in the two ESCC cell lines EC109 and EC9706 were assessed. The results demonstrated that the mRNA level of both PRR11 and SKA2 were significantly higher in EC9706 cells compared with EC109 cells. The protein expression of both PRR11 and SKA2 was also higher in EC9706 cells (Fig. 1C and D).

#### PRR11 and/or SKA2 knockdown inhibits the proliferation, migration, and invasion of ESCC

**Figure 2.**
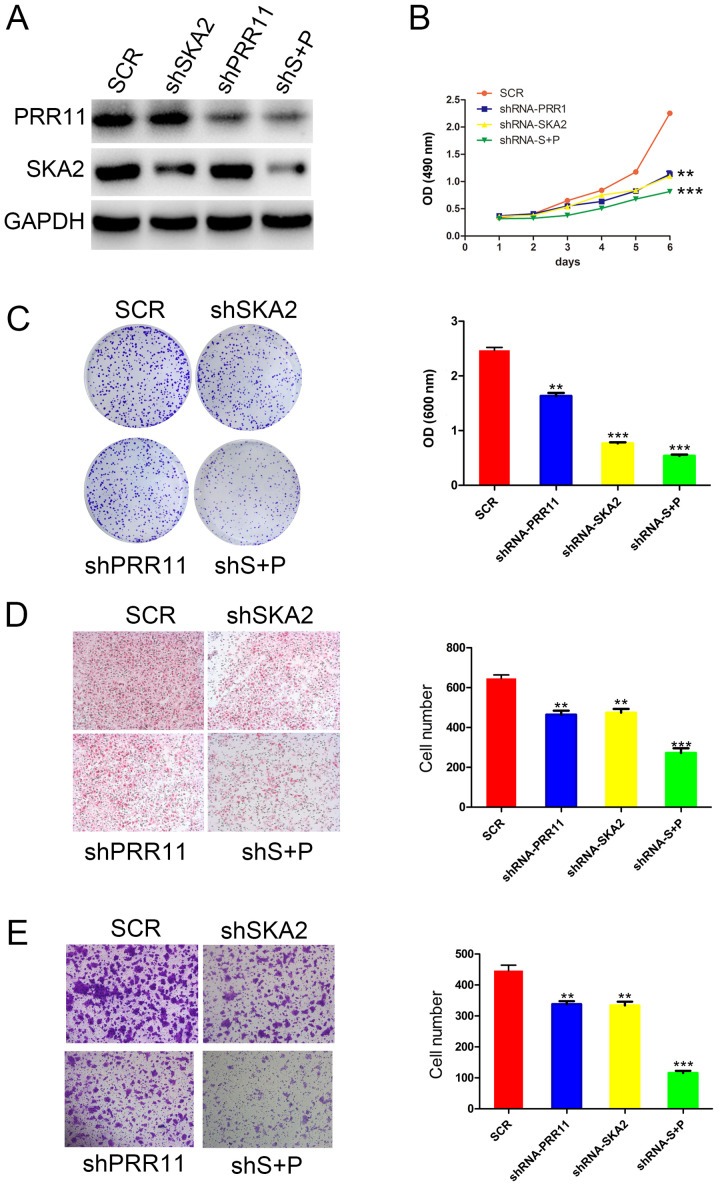
PRR11 or/and SKA2 knockdown inhibited proliferation, migratory and invasive capacities of ESCC cells. (A) Western blotting confirming PRR11 or/and SKA2 in EC9706 cells. (B) Effect of PRR11 or/and SKA2 knockdown on the proliferation of EC9706 cells assessed by MTT assay. (C) Effect of PRR11 or/and SKA2 knockdown on EC9706 cell proliferation assessed by crystal violet assay. (D) Effect of PRR11 or/and SKA2 knockdown on the migratory capacity of EC9706 cells assessed by Boyden chamber assay. (E) Effect of PRR11 or/and SKA2 knockdown on the invasive capacity of EC9706 cells assessed by transwell assay. Data were presented as mean ± standard deviation. **P<0.01 and ***P<0.001. OD, optical density; P and PRR11, proline-rich protein 11; S and SKA2, spindle and kinetochore associated 2; SCR, scramble; Sh, short hairpin.

To identify the function of the PRR11 and SKA2 gene pair in ESCC cells, specific shRNA targeting PRR11 and/or SKA2 were used in EC9706 cells. The efficiency of these knockdowns was confirmed by western blotting (Fig. 2A). Furthermore, the results from MTT and crystal assays demonstrated that transfection with shPRR11 or SKA2 significantly reduced EC9706 cell proliferation compared with untransfected cells. In addition, double transfection induced a more significant decrease in cell proliferation compared with single transfections (P<0.01 and P<0.001; Fig. 2B and C). In addition, as presented in Fig. 2D, the results from Boyden chamber assay demonstrated that the migratory ability of EC9706 cells was significantly decreased following PRR11 or SKA2 knockdown compared with untransfected cells (P<0.01 and P<0.001, respectively; Fig. 2D). Cell migratory capacity was also significantly more decreased following double knockdown of PRR11 and SKA2 compared with single transfections (P<0.001; Fig. 2C). Furthermore, the results from transwell assay indicated that the invasive capacity of EC9706 cells was significantly reduced following PRR11 and/or SKA2 knockdown (P<0.01 and P<0.001; Fig. 2E).

#### PRR11 and/or SKA2 overexpression promotes the proliferation, migration, and invasion of ESCC

**Figure 3.**
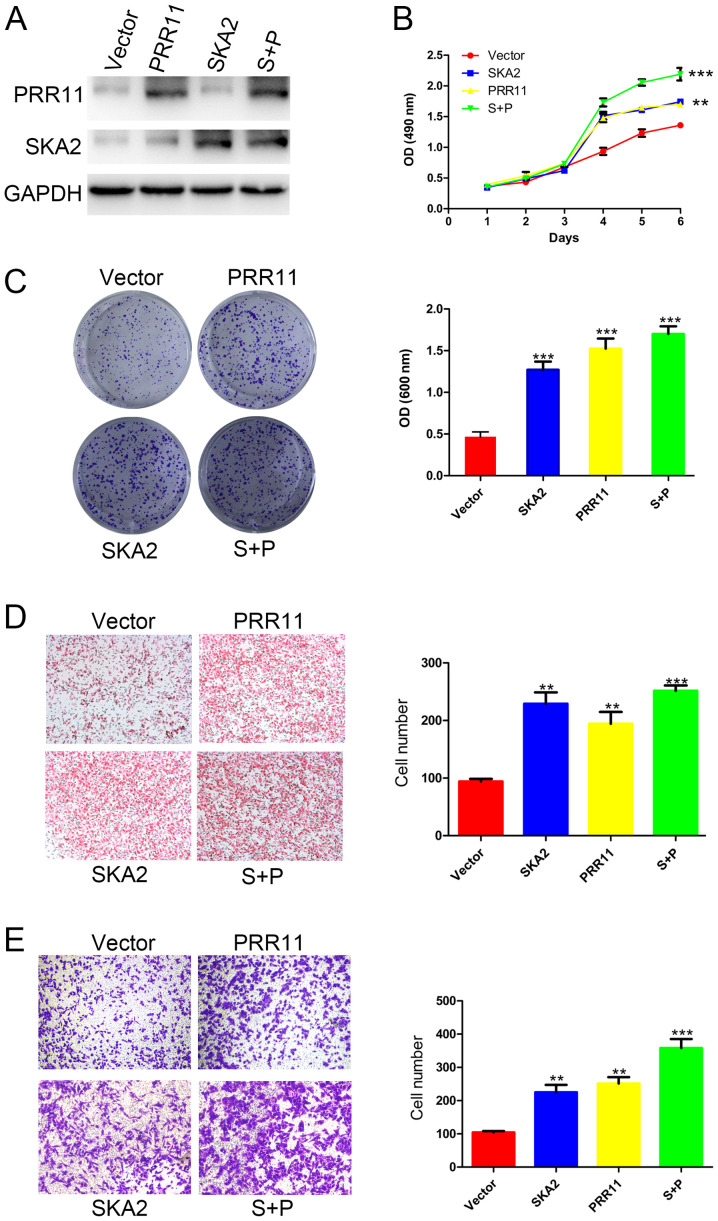
PRR11 or/and SKA2 overexpression promoted proliferation, migratory and invasive capacities of ESCC cells. (A) Western blotting confirming PRR11 or/and SKA2 overexpression in EC109 cells. (B) Effect of PRR11 or/and SKA2 overexpression on the proliferation of EC109 cells assessed by MTT assay. (C) Effect of PRR11 or/and SKA2 overexpression on EC9706 cell proliferation assessed by crystal violet assay. (D) Effect of PRR11 or/and SKA2 overexpression on the migratory capacity of EC109 cells assessed by Boyden chamber assay. (E) Effect of PRR11 or/and SKA2 overexpression on the invasive capacity of EC109 cells assessed by transwell assay. Data were presented as mean ± standard error. *P<0.05, **P<0.01 and ***P<0.001. OD, optical density; P and PRR11, proline-rich protein 11; S and SKA2, spindle and kinetochore associated 2; SCR, scramble; Sh, short hairpin.

PRR11 and/or SKA2 were overexpressed in EC109 cells by transfection. The successful overexpression of PRR11 and/or SKA2 was confirmed by western blotting (Fig. 3A). The results from MTT and crystal violet assays demonstrated a significantly increased cell proliferation following PRR11 or SKA2 overexpression compared with untransfected cells. In addition, the double transfection induced a more significant increase in cell proliferation compared with single transfections (P<0.01 and P<0.001; Fig. 3B and C). Furthermore, as presented in Fig. 3D, the results from Boyden chamber assay demonstrated that the migratory ability of EC109 cells was significantly increased following PRR11 or SKA2 overexpression compared with untransfected cells (P<0.01; Fig. 3D). Cell migratory capacity was more significantly increased following overexpression of PRR11 and SKA2 compared with single transfections (P<0.001; Fig. 3D). In addition, the results from transwell assay reported that overexpression of the gene pair significantly promoted the invasive ability of EC109 cells (P<0.01 and P<0.001; Fig. 3E).

#### PRR11 and SKA2 gene pair activates AKT and epithelial- mesenchymal transition (EMT) signaling pathways

**Figure 4.**
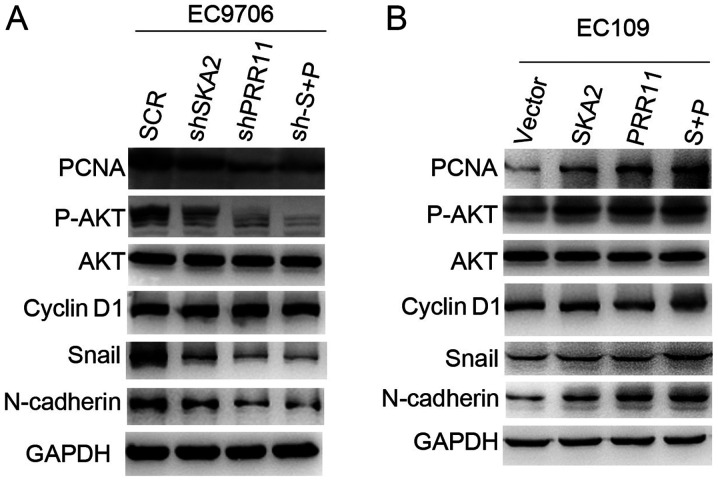
PRR11 or/and SKA2 overexpression promotes the AKT and EMT signaling pathways. (A) Effect of PRR11 or/and SKA2 knockdown on the expression of AKT, EMT-associated proteins and cell cycle-related proteins in EC9706 cells examined by western blotting. GAPDH was used as a loading control. (B) Effect of PRR11 or/and SKA2 overexpression on the expression of AKT, EMT-associated proteins and cell cycle-related proteins in EC109 cells examined by western blotting. GAPDH was used as a loading control. EMT, *epithelial-mesenchymal transition*; PRR11, proline-rich protein 11; SKA2, spindle and kinetochore associated 2.

In order to explore the underlying mechanism of the gene pair PRR11 and SKA2 on ESCC progression, the protein expression of p-AKT, total AKT, Snial and N-cadherin of EMT, and Cyclin D1 of cell cycle signal in EC9706 and EC109 cells by western blotting. The results demonstrated that expression levels of p-AKT, Snail and N-cadherin were reduced following PRR11 and SKA2 knockdown in EC9706 cells, and double transfection induced a more notable decrease in the expressions of these proteins compared with single transfections (Fig. 4A). Opposite effects were observed in EC109 cells overexpressing PRR11 and SKA2 (Fig. 4B). In addition, PRR11 and SKA2 overexpression or knockdown had no effect on Cyclin D1 expression. Furthermore, PRR11 and SKA2 overexpression increased the expression of the proliferation marker PCNA, whereas their knockdown decreased PCNA expression (Fig. 4A and B).

## Discussion

Over the past few decades, the prognosis of ESCC has been relatively poor in China, with the 5-year overall survival rate <20% and most patients died within 1 year of diagnosis (2). In addition, this disease lacks sensitive and specific early diagnosis method. Surgical and radiation therapies are the main treatments for ESCC; however, numerous patients eventually develop advanced stages of ESCC (4). The present study aimed therefore to identify potential intervention targets for ESCC.

Recent studies reported that the gene pair of PRR11 and SKA2 is involved in tumorigenesis and cancer progression. Zhu *et al* (9) reported that PRR11 overexpression facilitates ovarian carcinoma cell proliferation, migration, and invasion through activation of the PI3K/AKT/β-Catenin pathway. Furthermore, it was reported that the gene pair PRR11 and SKA2 is negatively regulated by p53 through nuclear factor Y in lung cancer cells (10). Also, PRR11 silencing leads to cell cycle arrest, suppresses colony formation, decreases cell proliferation and inhibits tumorigenic potential of lung cancer cells (19). In addition, the PRR11 and SKA2 gene pair has been hypothesized as a potential new target for the diagnosis and treatment of lung cancer (20). It has reported that overexpression of PRR11 could promote breast cancer progression by activating EMT (7). Qiao *et al* (6) demonstrated that proline-rich protein 11 silencing inhibits hepatocellular carcinoma growth and epithelial-mesenchymal transition through β-catenin signaling.

The present study demonstrated that PRR11 and SKA2 mRNA levels were significantly increased in ESCC tissues compared with adjacent normal tissues. Furthermore, cell proliferation, migratory and invasive capacities were significantly increased following PRR11 and SKA2 overexpression. In addition, PRR11 and SKA2 knockdown inhibited the proliferation, invasive and migratory capacities of ESCC cells. Subsequently, in order to investigate the underlying mechanism, this study demonstrated that PRR11 and SKA2 overexpression significantly activated the AKT signaling pathway and certain markers, including Snail and N-cadherin of EMT. AKT signaling pathway activation is implicated in the development of a numerous human cancers, including ESCC (21–23). Furthermore, EMT represents a critical event in the transition from early to invasive carcinomas, and it was demonstrated that N-cadherin upregulation is associated with poor prognosis and lower survival in patients with cancer (24–26). These findings are consistent with the results from the present study. To the best of our knowledge, this study was the first to demonstrate the involvement of PRRl1 and SKA2 in the progression of ESCC.

In summary, this study demonstrated that the gene pair PRRl1 and SKA2 may serve a crucial role in the proliferation, migratory and invasive abilities of ESCC cells. These results provided a better understanding of the underlying mechanisms of PRRl1 and SKA2 in ESCC tumor development, and PRRl1 and SKA2 may be considered as potential targets for the diagnosis and/or treatment of ESCC.

## Figures and Tables

**Table I.** Clinicopathological characteristics of the 30 patients included in the present study.

Variables	Number (%)
Sex	
Male	23 (76.7)
Female	7 (23.3)
Age, years	
<60	19 (63.3)
≥60	11 (36.7)
Smoking	
Yes	17 (56.7)
No	13 (43.3)
Drinking	
Yes	12 (40.0)
No	18 (60.0)
Tumor site	
Cervical	2 (6.7)
Upper	9 (30.0)
Middle	10 (33.3)
Lower	9 (30.0)
TNM stage	
IA	5 (16.7)
IB	9 (30.0)
IIA	7 (23.3)
IIB	5 (16.7)
IIIA	2 (6.7)
IIIB	2 (6.7)
Differentiation	
Well	6 (20.0)
Moderate	17 (56.7)
Poor	7 (23.3)

TNM stage (15), Tumor-Node-Metastasis.

## Data Availability

The datasets used and/or analyzed during the current study are available from the corresponding author on reasonable request.
